# Measuring the accuracy of genome-size multiple alignments

**DOI:** 10.1186/gb-2007-8-6-r124

**Published:** 2007-06-26

**Authors:** Amol Prakash, Martin Tompa

**Affiliations:** 1 Department of Computer Science and Engineering, University of Washington, Seattle, WA 98195-2350, USA; 2 Thermo BRIMS Center, Memorial Drive, Cambridge, MA 02139, USA; 3 Department of Genome Sciences, University of Washington, Seattle, WA 98195-2350, USA

## Abstract

Whole-genome alignments are invaluable for comparative genomics. Before doing any comparative analysis on a region of interest, one must have confidence in that region's alignment. We provide a methodology to measure the accuracy of arbitrary regions of these alignments, and apply it to the UCSC Genome Browser's 17-vertebrate alignment. We identify 9.7% (21 Mbp) of the human chromosome 1 alignment as suspiciously aligned. We present independent evidence that many of these suspicious regions represent misalignments.

## Abstract 2

A novel computational approach can assess the accuracy of genomic alignments, and reveals suspicious regions in the 17-vertebrate MULTIZ alignment available on the UCSC Genome Browser.

## Background

With the rapid sequencing of so many related genomes, comparative genomics has emerged as one of the most important areas of computational biology. The workhorse of comparative genomics is the multiple sequence alignment, particularly whole-genome multiple sequence alignments such as those provided by the UCSC Genome Browser [1]. These alignments are marvelous tools for anyone working in comparative genomics. More and more sophisticated analyses rely implicitly on the correctness of these alignments. For example, it is already standard practice to search for functional genomic elements (more precisely, those constrained by purifying selection) by scanning a whole-genome alignment, looking for regions that are better conserved across the species than expected [2-12].

When such methods find surprisingly well conserved sites across all aligned species, that portion of the alignment is likely to be correct. Conversely, in regions where the sequences are misaligned, these methods may fail to find conserved sites that exist. Even the designers of the alignment algorithms and genome browsers would not claim that the alignments are correct at all sites across entire genomes. How can users decide which portions of the alignment are trustworthy and which portions less so, particularly in noncoding regions?

We present a method to assess a whole-genome multiple sequence alignment, classifying it into well aligned and suspiciously aligned regions. Before carrying out any further analysis that relies on the alignment's correctness, such as those listed above, the user should be aware of possible misalignment in those regions classified as suspicious. In addition, efforts should be made to either realign the regions with suspicious alignments or increase the confidence in their current alignments by other evidence.

Without any well established methodology for estimating the quality of the whole-genome alignments, scientists have either trusted the alignments completely or developed their own filters. Such filters usually involve a conservation threshold (for example, a high phastCons conservation score [8,10]) and thus filter out most of the genomic sequence, limiting the scope of their analysis to only the regions that have high sequence similarity. Our work is the first that assigns alignment 'quality scores' to all portions of the whole-genome alignments. Using these, we have a measure of the level of trust in any region of the alignment, including those that have low sequence similarity. Thus, any comparative analysis can be carried out on much more of the genome than just those regions with high sequence similarity. Also, for the first time, we have a quantitative measure of how well a multiple alignment tool such as MULTIZ (the alignment tool used by the UCSC Genome Browser) performs on a whole-genome scale.

We note in this regard that the UCSC whole-genome multiple alignments are already annotated with phastCons conservation scores [9]. However, these scores serve a different purpose than our alignment quality scores: phastCons measures how well conserved each column of aligned residues is, under the assumption that the alignment is correct, that is, that aligned residues are indeed orthologous. The same statement applies to the measures 'binomial *p*-value' and 'parsimony *p*-value' proposed by Margulies *et al*. [5] and GERP [3] and Gumby [13] scores. In contrast, the purpose of our alignment quality scores is to assess the likelihood that the alignment is correct. So, for instance, a low phastCons score in an alignment segment that also has a poor alignment quality score does not necessarily mean poor conservation across the species, since the alignment may well be incorrect in this region. The same is true for the other four conservation scores mentioned above.

The statistics of local pairwise alignments [14,15] are well understood and have been extended to local multiple alignments [16]. These statistics are based on a few well conserved parts of the alignment, for example, protein domains, and report the statistical significance of these parts only. This cannot be used for our task on whole-genome alignments, because a whole-genome (or whole-chromosome) alignment will nearly always contain some very strongly conserved regions [2]. Thus, only these regions would be reported as statistically significant, with no information given about the remainder of the alignment. Unfortunately, there has been no methodology that measures the accuracy of every region of a multiple sequence alignment separately. (Yu and Smith [17] proposed a method based on hidden Markov models for doing this in pairwise alignments.) Solving this problem is critical in whole-genome alignments, because the search space for good alignments is huge due to genome rearrangements. Thus, there is a high probability that some regions will be misaligned even if most of the alignment is correct.

In previous work [16] we presented a tool, StatSigMA, to assess whether a multiple sequence alignment is contaminated with one or more unrelated sequences. In this work, we extend those ideas to the tool StatSigMA-w, which performs this assessment for every region of a multiple sequence alignment, and show its application to whole-chromosome alignments.

There are different ways that alignments can be incorrect, in the sense of not pairing all and only orthologous residues. There can be orthologies that are not aligned (false negatives of the alignment algorithm) and nonorthologies that are aligned (false positives of the alignment algorithm). StatSigMA-w is designed to measure the latter. These false positives can be further classified as 'small' errors (for example, one residue placed at the wrong end of a gap) or 'large' errors (for example, a region of 100 residues of one sequence aligned to regions of other sequences to which it is unrelated). Because StatSigMA is designed to detect contamination of a multiple sequence alignment by unrelated sequences, the focus of this work is on the latter, 'large' errors.

The UCSC Genome Browser provides whole-genome alignments for vertebrates, insects, and yeast. Of these, we evaluate the quality of the 17-vertebrate MULTIZ [18] alignment (to human genome assembly NCBI Build 36.1, UCSC hg18, March 2006) using StatSigMA-w. The 17 vertebrates are human, chimp, rhesus, rabbit, mouse, rat, dog, cow, armadillo, elephant, tenrec, opossum, chicken, frog, tetraodon, fugu, and zebrafish.

In the Results section we focus on the alignment of human chromosome 1 as a proof of concept. We identify 9.7% (21 Mbp) of this alignment as suspiciously aligned. We present independent evidence to support our claim that the suspicious regions may be misaligned. Overall, our work confirms that MULTIZ is doing an impressive job of aligning sequences. However, there are instances when it fails and these can be identified using StatSigMA-w.

## Results and discussion

### StatSigMA

The method used in this work extends our earlier tool StatSigMA [16], which assesses whether a multiple sequence alignment is contaminated with one or more unrelated sequences. There are several interesting problems in extending StatSigMA to assess the accuracy of all portions of a whole-genome alignment. We summarize the methodology of StatSigMA in this section and then describe its extension to StatSigMA-w in the subsequent sections.

Given a multiple alignment and a phylogeny relating its sequences, StatSigMA computes a *p*-value for each of several null hypothesis cases. There is one null hypothesis case for each branch *k *of the phylogeny. In the null hypothesis case *k*, branch *k *exhibits 'unrelated behavior', that is, the subalignments corresponding to the two subtrees separated by the removal of *k *are independent rather than homologous. The assumption made is that, after rejecting all these cases, we can infer that the multiple alignment shows all the sequences are related. The algorithm followed by StatSigMA is outlined below. These steps are described in detail elsewhere (A.P. and M.T., Assessing the discordance of multiple sequence alignments, unpublished work).

#### Algorithm followed by StatSigMA

Input: multiple sequence alignment and phylogeny.

1. For every branch *k *of the phylogeny do the following steps:

(a) Create the log likelihood scoring function corresponding to unrelated behavior on branch *k*.

(b) Using the scoring function, estimate Karlin-Altschul parameters *K*_*k*_, *λ*_*k*_, and *H*_*k *_[14].

(c) Using the scoring function, identify the maximal scoring segments of the input multiple alignment [19].

(d) Using *K*_*k*_, *λ*_*k*_, and *H*_*k*_, identify the set of maximal scoring segments resulting in the least *p*-value *p*_*k *_of the score [20].

2. Output *max*_*k *_*p*_*k *_as the 'discordance' of the multiple sequence alignment.

Analogous to a *p*-value, the discordance is between 0 and 1, and the lower its value, the better the alignment (in the sense of not being contaminated with unrelated sequences).

The log likelihood scores in the first step take into account the phylogeny. These scores are computed as follows. Suppose we have *S *sequences in a multiple alignment related by a phylogenetic tree *T *(having branch lengths). Let *γ*_1_, *γ*_2_, ..., *γ*_*S *_be the residues observed at a particular column of the multiple alignment. Suppose we want to test the hypothesis that there is unrelated behavior on branch *k*. Suppose the removal of branch *k *separates *T *into subtrees *t*_1 _(having residues *β*_1_, *β*_2_, ..., *β*_*i *_at the leaves) and *t*_2 _(having residues *β*_*i*+1_, *β*_*i*+2_, ..., *β*_*S *_at the leaves). Let *M *be the evolutionary model. Then, analogous to the Karlin-Altschul log likelihood score [14], the score for observing this column of the multiple alignment is as follows:

(1) $$sc_{k}(\gamma_{1},...,\gamma_{S}|T,M)=\log(\frac{\mathrm{Pr}(\gamma_{1},...,\gamma_{S}|T,M)}{\mathrm{Pr}(\beta_{1},...,\beta_{i}|t_{1},M)\mathrm{Pr}(\beta_{i+1},...,\beta_{S}|t_{2},M)})$$

We precompute this score for all tuples at the leaves of the tree. The various probability terms in Equation 1 are computed using the dynamic programming algorithm of Felsenstein [21].

### Appropriate phylogeny

Choosing the appropriate phylogeny is important for the accurate computation of *p*-values. In the case of pairwise alignments, without any information about the appropriate scoring matrix, BLAST [22] computes the *p*-value using multiple scoring matrices, and then corrects for multiple hypotheses. Using similar ideas, we use multiple trees to cover the variations in the rate of evolution across the genome. We start with the phylogeny estimated from four-way degenerate sites (that is, third codon positions) in the ENCODE [23] regions (Adam Siepel, personal communication), whose branch lengths correspond to neutral evolution. (This phylogeny is available on our worldwide web site [24].) We use three versions of this tree, one with the original branch lengths, one with branch lengths multiplied by 100 (to account for regions evolving faster than third codon positions), and one with branch lengths multiplied by 0.01 (to account for regions under purifying selection, for example, coding regions). Separate *p*-values are computed using each of the three trees, and the final score at a site is the minimum of the three, corrected for multiple hypotheses.

### Appropriate segmenting

The MULTIZ whole-genome alignment is built with human as the reference species. This alignment consists of chains of aligned blocks. The aligned blocks themselves may contain only a subset of the species. For example, for some human sequence, the aligned block may contain sequence from chimp and not from fugu. Human, being the reference species, is present in every block. As in StatSigMA [16], the null hypothesis case *k *refers to branch *k *of the phylogeny demonstrating unrelated behavior, and thus splitting the tree into two subtrees, *t*_1 _and *t*_2_, whose corresponding subalignments are independent. If a MULTIZ block does not contain any sequence from *t*_1 _or does not contain any sequence from *t*_2_, then that block is not considered for branch *k*.

Using StatSigMA's score function and the algorithm to find maximal scoring segments by Ruzzo and Tompa [19], we can identify all maximal, positively scoring segments in the whole-genome alignment. Each such segment can be a high scoring slice of a MULTIZ block or can overlap multiple MULTIZ blocks. Thus, it is possible that neighboring columns of a maximal scoring segment may contain different sets of species. For the rest of the paper, the word 'segments' refers to these maximal positively scoring segments. The score *sc*_*k*,*S *_of segment *S *is simply the sum, over the columns of *S*, of the log likelihood score of each column given by Equation 1. Here we make the assumption that the scoring function precomputed for branch *k *is accurate even when only a subset of species is aligned, so that we can add scores of columns that contain different sets of species.

### Appropriate context

The context for a local alignment is defined as the genomic sequences that were considered to create that alignment. For example, we may take genomic sequences of length 1 Mbp each from human and fugu, which may result in a local alignment of length 100 bp. Thus, the context for this 100 bp alignment is one million residues each for human and fugu.

Choosing the appropriate context is critical for the correct evaluation of *p*-values. A statistically significant alignment in a context of length *L *may be insignificant had the context length been 10*L*. (See Materials and methods for the exact dependence of the *p*-value on *L*.) In theory, as a multiple alignment tool proceeds to create an alignment, it can identify the context that it is using to output the alignment. However, it is unclear how to infer the original context from the completed alignment.

To solve this problem, we estimate the *p*-value of the score *sc*_*k*,*S *_ of segment *S *using all possible contexts *W *and then maximize over *W*; see Materials and methods for details of the *p*-value formulas. The reason for maximization over *W *is that, if even one context has a *p*-value greater than the threshold at which the null hypothesis is rejected, then the null hypothesis should not be rejected.

In order to evaluate the *p*-value in all possible contexts efficiently, we exploit strongly conserved genomic anchors. Throughout the genome, we expect to see very strongly conserved long segments (for example, certain coding exons). In fact, these segments are so highly conserved that their alignments to the orthologous regions in other species are statistically significant even if we compute the *p*-value of the score of this single segment in the context of the entire genome, that is, without any support from synteny. Once we have identified these anchors, for segments between anchors *A *and *B*, we need to consider only contexts that lie between *A *and *B*. This is because the *p*-values of the scores of the neighboring anchors are an upper bound on the *p*-value with respect to any context that contains them (see Materials and methods). Once we have computed the *p*-value of a segment's score, we can also treat that segment as an anchor for the later stages (for the purposes of upper bounding the *p*-value of nearby segments whose *p*-values are still to be computed). This gives rise to a divide-and-conquer algorithm that simultaneously defines appropriate contexts and estimates *p*-values for all regions of any multiple alignment (see Materials and methods for details of the algorithm).

### Identifying suspicious regions of the alignment

The *p*-value of a single site for a branch *k *is the *p*-value of the score of the segment that contains that site for the null hypothesis case *k*. After computing the *p*-value for every branch of the phylogeny and for every segment, we identify the branch (or branches, in case of a tie) having the greatest *p*-value for each site: this maximum *p*-value is the 'discordance' of that site. This method outputs the discordance of every aligned site, and the worst branch(es) for that site. Finally, at each site, the least discordance among the three trees (multiplied by 3 for Bonferroni correction) is the final discordance value. We take 0.1 as the threshold for identifying sites with high discordance, as discussed in the next section.

The scoring function of Equation 1 is based on an evolutionary model, including evolution of gaps as single residue insertions or deletions, as described in earlier work [16] (and A.P. and M.T., Assessing the discordance of multiple sequence alignments, unpublished work). Because such models reflect an incomplete understanding of evolution, they may cause the method described thus far to underestimate the statistical significance of certain regions, for example, those containing long insertions or deletions, or short low scoring alignments flanked by high scoring alignments. The latter problem is exacerbated by the fact that the extreme value distributon used to compute the *p*-values does not hold for very small contexts. Also, we are interested in studying longer regions that are misaligned rather than misalignments of a few residues. With these motivations, we consider only those regions that: are at least 50 bp in length; and have discordance at least 0.1 at all sites; with the same branch being the worst one, that is, this branch corresponds to the maximum *p*-value at each site. (This can result in overlapping regions corresponding to different branches, in the case of a tie for greatest *p*-value.) To eliminate regions that would be labeled insignificant due to long insertions and deletions, we filter out regions that contain more than 50% gap columns for either of the two subsets of species separated by the worst branch. The regions remaining are the ones that StatSigMA-w reports as having suspicious alignment. For the remainder of the paper, the term 'suspicious regions' refers to these remaining high discordance regions, and we denote the set of suspicious regions by ℜ. The suspicious regions that are attributable to any branch on the path from zebrafish (for example) to human are denoted by ℜ_zebrafish_; these are regions where zebrafish (and possibly other species) appear misaligned to human.

The decisions incorporated in the definition of ℜ are purposely conservative. Any region labeled 'suspicious' has at least 50 consecutive alignment columns for which a single branch exhibits unrelated behavior. There are no long insertions or deletions with respect to this branch, so the label of 'suspicious' is not attributable solely to the manner in which the evolution of gaps is modeled.

### Suspicious regions in chromosome 1

In this section, we present the results obtained by analyzing the MULTIZ 17-vertebrate alignment of chromosome 1 of the human genome. Chromosome 1 is 247 Mbp long, nearly 8% of the human genome. We took the MULTIZ alignment of this chromosome with the other 16 vertebrates, and computed *p*-values for all branches of the three phylogenies and for all segments.

**Figure 1:**
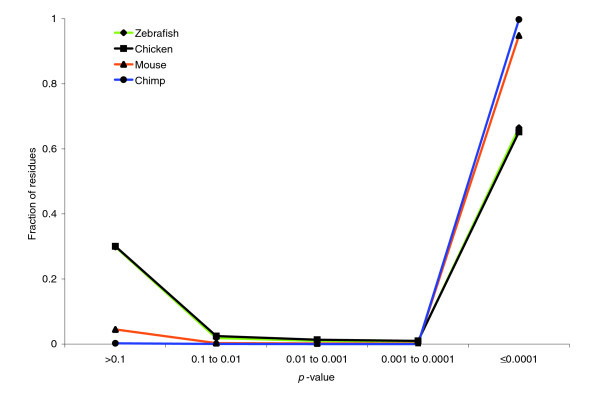
Distribution of alignment segment *p*-values. Fraction of residues in segments of length at least 50 bp plotted against the *p*-value of that segment's score, for the branches of the phylogeny incident on zebrafish, chicken, mouse, and chimp. The zebrafish and chicken graphs are so close as to be nearly indistinguishable.

For four representative branches, Figure 1 plots the fraction of sites in maximal scoring segments of length at least 50 bp against the *p*-value of that segment's score. As can be seen, the graphs are bimodal, that is, most columns either have *p*-value greater than 0.1 or less than 10^-4^. The graphs show that a threshold of 0.1 can be used to differentiate low *p*-value segment scores from high *p*-value segment scores, and this classification would hardly change if the threshold were instead 10^-2 ^or 10^-4^.

Table 1 provides a breakdown of the residues of human chromosome 1 in ℜ with respect to the other species. For example, 28.5% of the human residues aligned to zebrafish have a suspicious zebrafish alignment (that is, belong to ℜ_zebrafish_). Considering the human alignments containing mouse, approximately 3.3% have a suspicious alignment with mouse. In general, the nonprimates each have 1-5 Mbp in suspicious regions, and the percentage of residues in suspicious regions increases (approximately) with increasing phylogenetic distance from humans. This suggests increasing difficulty of correct alignment as this distance increases. In total, ℜ contains 185,452 regions, varying in size from 50 bp to 3,121 bp, with mean 115 bp. The total number of residues contained in ℜ is more than 21 Mbp and accounts for 9.7% of the aligned human chromosome 1.

**Table 1:** Suspicious regions in the human chromosome 1 alignment

Species	Aligned residues		Residues in suspicious regions		Suspicious regions	Residues with high phastCons score in suspicious regions	
Chimp	211,636,048	(86%)	7,722	(0.004%)	103	174	(2.3%)
Rhesus	198,482,753	(80%)	32,789	(0.02%)	358	214	(0.7%)
Rabbit	87,544,213	(35%)	2,053,030	(2.3%)	24,210	24,037	(1.2%)
Mouse	92,668,918	(37%)	3,033,524	(3.3%)	36,594	25,175	(0.8%)
Rat	87,532,024	(35%)	3,141,584	(3.6%)	37,231	34,373	(1.1%)
Dog	132,538,114	(54%)	1,661,141	(1.3%)	19,277	8,383	(0.5%)
Cow	124,582,989	(50%)	1,524,553	(1.2%)	18,020	6,563	(0.4%)
Armadillo	81,991,879	(33%)	1,451,731	(1.8%)	17,306	25,172	(1.7%)
Elephant	88,888,989	(36%)	2,047,450	(2.3%)	23,089	30,143	(1.5%)
Tenrec	67,619,223	(27%)	2,495,171	(3.7%)	26,697	26,611	(1.1%)
Opossum	31,534,309	(13%)	4,657,853	(14.8%)	41,302	120,445	(2.6%)
Chicken	9,106,790	(4%)	2,363,996	(26.0%)	18,397	235,152	(9.9%)
Frog	5,916,044	(2%)	1,432,427	(24.2%)	10,947	219,613	(15.3%)
Tetraodon	5,167,534	(2%)	1,765,895	(34.2%)	9,508	240,568	(13.6%)
Fugu	4,630,234	(2%)	1,167,357	(25.2%)	8,437	169,851	(14.6%)
Zebrafish	5,855,213	(2%)	1,669,490	(28.5%)	10,963	324,159	(19.4%)
All species	219,711,964	(89%)	21,334,060	(9.7%)	185,452	1,127,826	(5.3%)

Aligned portions of the 247 Mbp of human chromosome 1 with respect to each of the other 16 vertebrates. Column 2 shows the total number of residues of human chromosome 1 that are aligned to the other species, together with its percentage of 247 Mbp, the size of human chromosome 1. Column 3 shows the total number of residues that are in suspicious regions, together with its percentage of the corresponding number in column 2. The number of suspicious regions is shown in column 4. Column 5 shows the total number of residues that are in suspicious regions and have a phastCons score greater than 0.5, together with its percentage of the corresponding number in column 3.

The last column of Table 1 provides the number and percentage of residues in ℜ that have a phastCons score greater than 0.5. The UCSC phastCons [9] track measures the level of conservation of any site in an alignment, and a score greater than 0.5 means that the site is more likely to be in the conserved state than the nonconserved state. Considering, for example, the human residues aligned to zebrafish, it can be seen that 19% of the residues in ℜ_zebrafish _have high phastCons scores. In general, the high percentages for the nonmammals in the last column are likely to correspond to regions where the mammals are well conserved but the given nonmammal may be misaligned. We did a separate test of phastCons on synthetic data, using the same parameter values that were used to compute the conservation score for the 17-vertebrate MULTIZ alignment. If all but one sequence are identical, and the remaining sequence is less than 50% identical to the others, phastCons can still report a very high conservation score at every site, including those where the last sequence is not identical. This is additional evidence that phastCons is not intended to identify misalignments.

**Figure 2:**
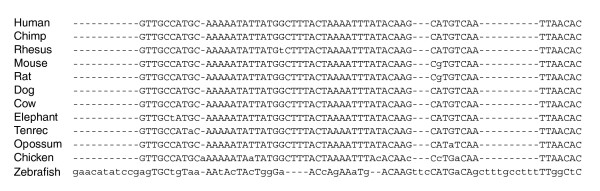
Sample suspicious zebrafish alignment. The MULTIZ alignment block at human coordinates chr1: 87,801,892-87,801,950, covering 59 bp of human sequence. Lower case letters indicate disagreement with the human sequence.

Figure 2 provides an example of a MULTIZ alignment block in ℜ_zebrafish_. Lower case letters in this multiple alignment indicate disagreement with the human sequence. StatSigMA-w's reported discordance is 1 at every site in this block, the branch incident on zebrafish being responsible for this high value. In contrast, the phastCons conservation score is 1 at every human site in this block, despite the lack of conservation in the zebrafish sequence. (Recall that a StatSigMA-w discordance of 1 suggests misalignment, whereas a phastCons score of 1 indicates strong conservation.) This exemplifies the discussion in the previous paragraph that a high phastCons score does not imply correct alignment. In this region, the high phastCons score is due to very strong conservation not only among the mammals, but including chicken as well.

### Independent evidence of misalignment

**Figure 3:**
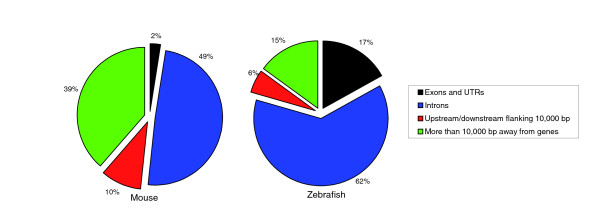
Pie charts showing the genomic distribution of suspicious regions for zebrafish and mouse. UTR, untranslated region.

Figure 3 presents a pie chart showing the distribution of genomic locations of the suspicious regions in ℜ_zebrafish _and ℜ_mouse_. These are based on the annotations of the aligned human regions available on the UCSC Genome Browser. The majority of the suspicious regions lie far from genes or in introns. For zebrafish, a small percentage (16.9%) are aligned to human exons. This is in contrast to the fact that nearly 35% of the chromosome 1 alignments containing zebrafish lie in human exons. Only 2.4% of ℜ_mouse _regions are aligned to human exons, in contrast to the fact that nearly 6% of the chromosome 1 alignments containing mouse lie in human exons. This is consistent with the intuition that misalignments will be rarer in exons than in noncoding regions. The overlap of suspicious regions with coding sequences does point out, however, that misalignments can be misleading, especially if the alignment is being used to infer functional annotation in one species from another.

**Figure 4:**
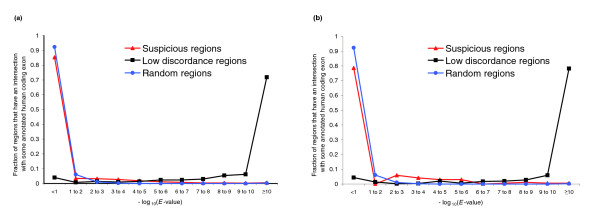
Distribution of BLASTX *E*-values in coding regions. **(a) **Distribution of BLASTX *E*-values for ℜ_zebrafish _and low discordance (≤10^-10^) zebrafish alignment regions intersecting human coding exons. The distribution is also plotted for random zebrafish nucleotide sequences. **(b) **The analogous distributions for mouse.

As described in the previous paragraph, 16.9% of suspicious regions in ℜ_zebrafish _overlap annotated human exons. Of these, 770 regions in ℜ_zebrafish _overlap the coding exons of 562 human genes. This suggests that the alignment is incorrectly aligning a human coding sequence and a sequence from zebrafish. To test this hypothesis, we took each human coding sequence aligned to a region in ℜ_zebrafish_, translated it using its annotated reading frame to yield its amino acid sequence, and used this as a BLASTX query against the aligned zebrafish nucleotide sequence. BLASTX translates the zebrafish nucleotide sequence in all six reading frames, aligns each of them to the human amino acid sequence, and reports the best *E*-value among all six pairwise alignments. Figure 4a plots the distribution of these *E*-values for ℜ_zebrafish_. To contrast this to the distribution for well aligned sequences, we took those parts of the alignment that include zebrafish, have discordance at most 10^-10^, and intersect some human coding exon. The distribution of BLASTX *E*-values for these parts is also plotted in Figure 4a. Finally, the distribution when the zebrafish sequence is replaced by a random zebrafish nucleotide sequence is also shown in Figure 4a. This figure demonstrates that the regions in ℜ_zebrafish _look nearly as badly aligned as random sequences, whereas the regions in the low discordance zebrafish alignments have much lower *E*-values. The analogous graphs for ℜ_mouse _are shown in Figure 4b. Figure 4 provides strong evidence that many of the regions in ℜ_zebrafish _and ℜ_mouse _are indeed misaligned.

The data underlying Figure 4 can be used to obtain an estimate of the false positive rate of StatSigMA-w's prediction of suspicious regions. If we make the simplistic assumption (based on where the low discordance and suspicious curves cross in Figure 4a) that a BLASTX *E*-value at most 10^-4 ^indicates a correct alignment, then of the 770 predicted zebrafish suspicious regions aligned to a human exon, only 5.3% are in fact correctly aligned (compared to 93% of the low discordance regions).

In a more global sense, though, the MULTIZ coding alignments are better than Figure 4 suggests. When we repeat the BLASTX experiment on the entire annotated human exon rather than just the exon fragment aligned to the ℜ_zebrafish _region, the BLASTX *E*-value graph looks much more like the low discordance graph of Figure 4a. This suggests that MULTIZ usually aligns a homologous zebrafish exon, but misaligns the fragments of these exons that are in ℜ_zebrafish_.

**Figure 5:**
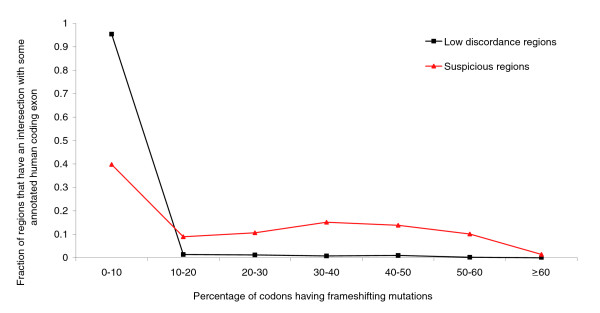
Percentage of frameshifted codons. Distribution of ℜ_zebrafish _and low discordance (≤10^-10^) zebrafish alignment regions intersecting some annotated human coding exons, plotted against the percentage of frameshifted zebrafish codons (in the region aligned to the human exon).

To analyze these exonic regions using a different criterion, we searched the aligned zebrafish sequence in all three forward reading frames to compute the percentage of frameshifted codons (because of many insertions and deletions in the alignment). We used the reading frame that led to the least percentage of frameshifted codons. Figure 5 plots the distribution of this percentage for the regions in ℜ_zebrafish_. To contrast this to the distribution for well aligned sequences, we took those parts of the alignment that include zebrafish, have discordance at most 10^-10^, and intersect some human coding exon. This distribution is also plotted in Figure 5. Like Figure 4, Figure 5 clearly shows that the two distributions are very different, and the suspicious regions have much more dubious alignments than the low discordance regions.

### Results on simulated data

In order to measure further the sensitivity and specificity of StatSigMA-w's predictions of suspicious regions, we also performed experiments on simulated data. Such experiments have the advantage that we know exactly what is correctly aligned and what is misaligned. The simulated data were provided by Mathieu Blanchette (personal communication). They consist of a set of 25 correct multiple sequence alignments, each of length approximately 100 Kb. Each alignment results from the concatenation of two data sets obtained by simulation of evolution according to the procedure described by Blanchette *et al*. [18] and summarized as follows. Each simulation begins with a random ancestral sequence and lets it evolve along the branches of a given phylogeny until it produces sequences at the leaves. The phylogeny used is that for the nine mammals human, chimp, baboon, mouse, rat, dog, cat, cow, and pig. By keeping track of the mutations performed, it is straightforward to produce the true alignment of orthologous residues and gaps. These are the alignments from which our experiments began.

From each of these alignments we discarded all the gaps and aligned the resulting sequences using TBA. (TBA is a generalization of MULTIZ in which there is no special reference sequence. Both algorithms are described in the same paper [18].) By comparing this TBA alignment to the true alignment, we determined exactly where TBA misaligned residues, in the sense that such a residue in human is aligned to a nonorthologous residue in another species. Analogous to the definition of a suspicious region, define a 'misalignment region' as any contiguous set of 50 or more columns in the TBA alignment in which a single species' residues are misaligned to human in every column. Regions with more than 50% gap characters in the misaligned species were ignored.

**Figure 6:**
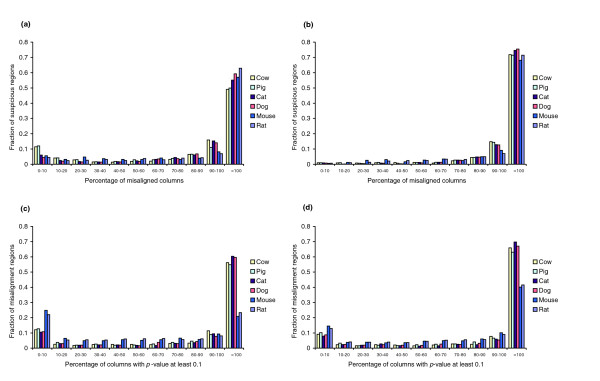
Specificity and sensitivity for simulated data. Results averaged over 25 simulated data sets, each of size approximately 100 Kb, that are aligned by TBA. **(a) **Specificity with respect to the same species: for each suspicious region reported by StatSigMA-w, the histogram shows the percentage of its columns for which the species reported as suspicious is actually misaligned by TBA. **(b) **Specificity with respect to any species: for each suspicious region reported by StatSigMA-w, the histogram shows the percentage of its columns for which any species is actually misaligned by TBA. **(c) **Sensitivity with respect to the same species: for each misalignment region, the histogram shows the percentage of its columns for which StatSigMA-w reports a *p*-value at least 0.1 for a branch attributable to the misaligned species. **(d) **Sensitivity with respect to any species: for each misalignment region, the histogram shows the percentage of its columns for which StatSigMA-w reports a *p*-value at least 0.1 for any branch.

We next submitted the TBA alignment to StatSigMA-w, which computed *p*-values and suspicious regions as described above. Our goal was to assess the specificity (fraction of suspicious regions actually misaligned) and sensitivity (fraction of misalignment regions reported as suspicious) for these StatSigMA-w predictions. The results are given in the four histograms of Figure 6. These histograms show averages over the 25 simulated data sets. Because TBA rarely misaligned chimp or baboon with human, histograms are given only for the other six species.

Figure 6a,b presents specificity results, reporting information about the distribution of suspicious regions. For each suspicious region reported by StatSigMA-w, we measured the percentage of columns of that region that are actually misaligned. This percentage is shown on the horizontal axis. In Figure 6a, the misalignment is required to be in the same species as a species responsible for the suspicious region; that is, if the suspicious region is attributable to branch *k*, then *k *must lie on the path from the misaligned species to human. In Figure 6b, the misalignment could be in any species. The reason for our interest in the latter is that it is sometimes the case that a misalignment in pig (say) may cause StatSigMA-w to report that cow appears suspiciously aligned, particularly if the cow sequence is sufficiently diverged from human. In any case, drawing attention to any misalignment by labeling it as suspicious should be helpful to the alignment user.

Figure 6c,d presents analogous sensitivity results, reporting information about the distribution of misalignment regions. For each misalignment region, we measured the percentage of columns of that region for which StatSigMA-w reports a *p*-value at least 0.1. In Figure 6c, the high *p*-value must be for a branch separating the misaligned species from human. In Figure 6d, the high *p*-value can be for any branch.

The results shown in Figure 6 are quite encouraging. A very large fraction of reported suspicious regions have at least 90% of their columns misaligned and, conversely, for a very large fraction of misalignment regions StatSigMA-w reports at least 90% of their columns to have high *p*-values.

## Conclusion

In this work, we analyzed the 17-vertebrate MULTIZ alignment of human chromosome 1. On the whole, MULTIZ is doing a good job of aligning orthologous sequences. However, 9.7% of the alignment is identified as suspiciously aligned by our method. We present independent evidence that many of these suspicious regions represent misalignments. This evidence is for coding sequences, which should be the simplest to align, and thus can be treated as a lower bound on the percentage of misalignments. Extrapolating to the entire genome, 3.2 Gbp × 9.7% = 310 Mbp of the alignment may be suspicious and should be reexamined.

It is possible that some of the regions that we report as suspiciously aligned may indeed be correctly aligned. The sequence similarity may be too low to establish confidence in the alignment, but it may still be correct. In such cases we need additional evidence to trust the alignment and use it as the basis for comparative sequence analyses.

As shown in Table 1, only a fraction of human chromosome 1 was actually aligned to other vertebrates. For example, approximately 2% of human chromosome 1 is aligned to zebrafish. While there are sequences in human for which it will be very hard to find the correct orthologous zebrafish sequence (in fact, orthologous zebrafish sequences may not exist), an alignment tool other than MULTIZ may be able to align sequences that MULTIZ could not. Using StatSigMA-w we can take the alignment created by any tool and evaluate its discordance. Thus, we can leverage the strengths of all tools and significantly align as much of the human genome as possible.

While this work deals with analyzing whole-genome alignments, StatSigMA-w can also be used to analyze any alignment to measure the confidence in various parts of the alignment. This can be useful, for example, if we suspect there are several domains in a protein multiple alignment, and the high conservation of some is causing the whole alignment to look good. Until now there was no method to measure whether the individual domains (other than the well conserved ones) are significantly aligned or not. Using StatSigMA-w, we can assign a discordance value to all parts of the alignment.

The StatSigMA-w track for the UCSC Genome Browser available on our web site [24] can be used as a quality measure of the alignment at any locus of interest on human chromosome 1. Source code for StatSigMA-w is available on request from the authors.

## Materials and methods

### *p*-value of a segment in a given context

Let *W *be any genomic context that contains the segment *S*; *sc*_*k*,1_, *sc*_*k*,2_, ..., *sc*_*k*,m _are the scores (in decreasing order) of the other segments in *W *with respect to branch *k*. As described earlier [16] (and A.P. and M.T., Assessing the discordance of multiple sequence alignments, unpublished work), the normalized segment scores for these segments are *sc*'_*k*,*i *_= *λ*_*k*_*sc*_*k*,*i *_- ln(*K*_*k*_(|*W*| - *H*_*k*_)^2^), where *K*_*k*_, *λ*_*k*_, and *H*_*k *_are the Karlin-Altschul parameters for the null hypothesis case *k*.

For any 0 ≤ *r *≤ *m*, define $total_{k,r}={\sum_{i=1}^{r}sc^{\prime }}_{k,i}-ln(r!)$[15]. Then, for the null hypothesis case *k*, the *p*-value of *sc'*_*k*,*S *_in context *W *is:

(2) $$p-value(sc{'}_{k,S}|W,k)=\overset{m}{\underset{r=0}{\min}}(p-value(total_{k,r}+sc{'}_{k,S}|W,k,r)\times 2^{r+1}).$$

The formula to compute the *p*-value of *total*_*k*,*r *_+ *sc'*_*k*,*S *_given context *W *is presented elsewhere (A.P. and M.T., Assessing the discordance of multiple sequence alignments, unpublished work). Using ideas from Altschul [20], Equation 2 minimizes the *p*-value over all values of *r *and corrects for multiple hypotheses (multiplication by 2^*r*+1^).

Now to compute the *p*-value for segment *S *over all possible contexts *W *containing *S*:

(3) $$p-value(sc{'}_{k,S}|k)=\underset{W}{\max}(p-value(sc{'}_{k,S}|W,k)).$$

The reason for maximization in Equation 3 is that, if even one context has a *p*-value greater than the threshold at which the null hypothesis is rejected, then the null hypothesis should not be rejected. The next section shows how to estimate the *p*-values of Equation 3 without explicitly considering every possible context *W*.

### Algorithm to limit contexts and estimate *p*-values

Since the MULTIZ alignments are human-referenced, the genomic location of the human sequence contained in a segment uniquely identifies that segment. For purposes of notation, let (*S*_*i*,*start*_, *S*_*i*,*end*_) be the human coordinates that identify segment *S*_*i*_.

The ideas outlined in the Results section give rise to the following divide-and-conquer algorithm that simultaneously defines appropriate contexts and estimates *p*-values for all regions of any multiple alignment.

Step 1. Consider all positively scoring segments in decreasing order of their scores.

Step 2. For segment *S*, compute its *p*-value *p*_*S *_taking the entire genome as the context and ignoring other segment scores.

Step 3. Repeat Step 2 for all the highest scoring segments *S *as long as *p*_*S *_< 10^-10^.

Step 4. For segment *S*, identify its closest left segment *S*_*L *_and right segment *S*_*R *_in the human genome for which *p*-values have been computed. If the worst context of *S*_*L *_does not include *S*, let *L *be the leftmost segment of the worst context of *S*_*L*_, otherwise *L *= *S*_*L*_. Similarly, if the worst context of *S*_*R *_does not include *S*, let *R *be the rightmost segment of the worst context of *S*_*R*_, otherwise *R *= *S*_*R*_. Let the positively scoring segments between *L *and *R *(including *L *and *R*) be *S*_1_, *S*_2_, ..., *S*_*m*_, occurring in this order in the alignment.

Step 5. Consider any segments *S*_*i *_and *S*_*j *_(where *i *≤ *j*), such that *S *is between *S*_*i *_and *S*_*j*_. Let the context between *S*_*i*,*end *_and *S*_*j*,*start *_be *W*_*ij*_. Using Equation 2, compute *p*_*i*,*j *_= *p*-value(*sc'*_*k*,*S *_| *W*_*ij*_, *k*).

Step 6. Repeat Step 5 for all possible *i *and *j *such that *i *≤ *j *and *S *is between *S*_*i *_and *S*_*j*_. Let *p*_*LR *_be the maximum value of *p*_*i*,*j *_over all such *i *and *j*.

Step 7. The estimated (Equation 3) *p*-value of segment *S *is max(*p*_*LR*_, $p_{S_{L}}$, $p_{S_{R}}$), where $p_{S_{L}}$ and $p_{S_{R}}$ are the already computed *p*-values of *S*_*L *_and *S*_*R*_, respectively. Record the leftmost and rightmost segments in the worst context of *S*, for subsequent iterations of Step 4.

Step 8. Repeat Steps 4 to 7, until *p*-values have been computed for all positively scoring segments. Assign *p*-value 1 to any negatively scoring segments.

A point to note here is that, if a segment has a *p*-value *p *for a context *W*, then extending *W *to reach the nearest segment boundaries can only increase the *p*-value. Thus, for identifying the context with the greatest *p*-value in Step 5, we need only consider those contexts that lie on segment boundaries.
